# Association between knowledge of electronic cigarettes and HTPs and intentions to use among university students: A cross-sectional study

**DOI:** 10.18332/tid/224358

**Published:** 2026-07-07

**Authors:** Canan Arslan

**Affiliations:** 1 Department of Medical Services and Techniques, Plato Vocational School, İstanbul Topkapı University, İstanbul, Türkiye

**Keywords:** electronic cigarette, HTPs, knowledge level, intention to use, young adults

## Abstract

**INTRODUCTION:**

Electronic cigarettes and heated tobacco products (HTPs) have become increasingly visible among young adults. This study examines the association between university students’ level of knowledge about these products and their intention to use them.

**METHODS:**

This cross-sectional study was conducted among 382 university students aged 18–25 years in Türkiye. Participants were recruited using a convenience sampling method. Knowledge of electronic cigarettes and HTPs was assessed using true, false, or ‘I don’t know’ items. Intention to use was measured with Likert-type items. Group differences were analyzed using the Mann–Whitney U test, and associations were examined using Kendall’s tau-b correlation coefficient. Multivariable ordinal logistic regression analyses were performed to adjust for potential confounding factors. Statistical significance was set at 0.05.

**RESULTS:**

Awareness of electronic cigarettes was high (94.2%), whereas current use was low (0.8%). For HTPs, awareness was 34.8%, and current use was 3.7%. The mean knowledge scores were 3.25 ± 2.22 for electronic cigarettes and 2.22 ± 2.51 for HTPs. Students who indicated that they might consider trying an HTP if offered by a friend had higher knowledge scores (p=0.047). Weak but statistically significant positive correlations were observed between knowledge of electronic cigarettes and intention to use within the next six months (τ-b=0.143; p<0.001), as well as overall propensity to use (τ-b=0.165; p<0.001). However, in multivariable analyses, knowledge was not independently associated with intention to use either electronic cigarettes or HTPs, while conventional cigarette use and peer use were significantly associated with intention outcomes.

**CONCLUSIONS:**

Knowledge level was not independently associated with intention to use electronic cigarettes or HTPs. Knowledge alone may not be sufficient to explain differences in intentions among young adults, and behavioral and social factors may play a more prominent role.

## INTRODUCTION

Tobacco use remains a leading cause of preventable diseases and premature deaths worldwide. The World Health Organization (WHO) reports that tobacco-related diseases account for more than seven million deaths annually^1^. While conventional cigarette use has decreased in some regions, the nicotine market is changing rapidly, particularly with the growing use of alternative products such as electronic cigarettes (e-cigarettes) and heated tobacco products (HTPs)^2,3^. In recent years, the use of these products has increased, particularly among adolescents and young adults, as they are presented as alternatives to conventional cigarettes. Misunderstandings about the nicotine content of e-cigarettes and their addictive potential may contribute to the perception that these products are less harmful^4^.

Similarly, HTPs are marketed as modern and low-risk alternatives, and such marketing has been associated with increased use, posing challenges for tobacco control efforts^5^.

Perceptions and misconceptions about e-cigarettes and HTPs play an important role in their increasing use among adolescents and young adults. Misconceptions about the safety of these products persist, despite evidence that they contain nicotine and other harmful constituents^6,7^. Studies among university students indicate that knowledge regarding the health risks of electronic cigarettes is insufficient and that there is a widespread belief that they are less harmful than conventional cigarettes^2,8^. Low risk perception, social influences, and product visibility are associated with increased intention to use these products among young adults^9^.

Similarly, perceptions regarding HTPs are shaped primarily by marketing and social narratives rather than scientific evidence. The presentation of HTPs as ‘cleaner’, ‘less odorous’, and ‘more socially acceptable’ than conventional cigarettes may increase experimentation and intention to use, particularly in peer environments. Studies indicate that a substantial proportion of young adults perceive HTPs as less harmful than conventional cigarettes, despite limited knowledge of their actual harms^10-12^. These findings suggest that the relationship between knowledge and intention to use may be complex and non-linear for both electronic cigarettes and HTPs.

Previous studies have examined awareness, perceptions, and use of electronic cigarettes or HTPs separately. However, evidence on the association between knowledge of these products and intention to use among university students remains limited. Understanding this relationship is important for tobacco control and the development of preventive strategies targeting young adults. This cross-sectional study, grounded in the Knowledge–Attitude–Practice (KAP) framework and the Theory of Planned Behavior (TPB)^13,14^, examines the association between university students’ knowledge of electronic cigarettes and HTPs and their intention to use these products.

## METHODS

### Study design

This study was a cross-sectional investigation conducted to examine the association between university students’ level of knowledge about electronic cigarettes (e-cigarettes) and HTPs and their intention to use these products. Data were collected between November and December 2025 using a structured self-administered online questionnaire. The study sample consisted of university students enrolled in associate and undergraduate degree programs across Türkiye, including both public and private universities. Participants were recruited using a non-probability convenience sampling strategy. The study was reported in accordance with the STROBE checklist (Supplementary file).

The sample size estimation was based on Cohen’s (1988) guidelines for effect sizes. Assuming a small-to-moderate effect size (Cohen’s d=0.30), a significance level of 0.05 (α=0.05), and 80% power (1-β=0.80), the minimum required sample size was calculated as 176 participants to detect a statistically significant effect. Considering potential subgroup analyses and the possibility of missing or excluded responses, the target sample size was set at 250–300 students.

### Inclusion and exclusion criteria

The inclusion criteria were: 1) being aged 18–25 years, 2) being enrolled in an associate or undergraduate degree program; and 3) providing informed consent. Participants who completed less than 80% of the questionnaire, provided inconsistent responses, or had participated in the pilot study, were excluded from the analysis.

### Data collection tools

The questionnaire used in this study was developed by the researcher for the purposes of this study, drawing on the Knowledge–Attitude–Practice (KAP) framework and the theory of planned behavior. Knowledge items were presented as factual true/false statements, with an additional ‘I don’t know’ option included to minimize guessing bias. Intention-to-use items were measured using five-point Likert-type scales to reflect their subjective nature. The questionnaire did not include any brand-specific or leading statements. The researcher-developed questionnaire consisted of the following sections.

*Sociodemographic characteristics*

This section included items assessing participants’ age, sex, year of study, academic department, type of university, geographical region, conventional cigarette use, and peer influence.

*Product awareness and use*

This section included items assessing awareness, experimentation, current use status (categorized as ‘Yes, currently’, ‘Tried but quit’, or ‘Never used’), and past 30-day use of electronic cigarettes and HTPs.

*Knowledge assessment*

Knowledge of electronic cigarettes and HTPs was assessed using items answered in a true/false/‘I don’t know’ format. The electronic cigarette section consisted of seven items, and the HTPs section consisted of eight items. Each correct response was coded as 1, whereas incorrect and ‘I don’t know’ responses were coded as 0. Total knowledge scores were calculated by summing the number of correct responses. Accordingly, electronic cigarette knowledge scores ranged from 0 to 7, and HTPs knowledge scores ranged from 0 to 8.

*Intention to use*

Intention to use electronic cigarettes and HTPs was assessed separately for each product using items with a five-point Likert-type response format.

*Susceptibility*

Among participants who had never used the respective product, susceptibility was assessed by evaluating the likelihood of trying the product.

### Contact validity, clarity, and pilot testing

The relevance and clarity of the knowledge items were evaluated using the Content Validity Index (CVI), based on assessments by five experts with experience in the field. Revisions were made where necessary. To assess clarity and comprehensibility, cognitive interviews were conducted with 15 students from the target population. A pilot study was conducted with 30 students to evaluate the feasibility of the questionnaire, including item clarity and completion time. Data obtained from the pilot study were not included in the main analyses. The internal consistency of the knowledge scales was assessed using Cronbach’s alpha, indicating good reliability for both the e-cigarette (α=0.812) and HTPs (α=0.870) knowledge scales.

### Data collection procedure

Data were collected via an online survey platform. The first page of the questionnaire provided information about the study’s purpose, scope, voluntary participation, confidentiality, and the right to withdraw at any time. Participants provided electronic informed consent before proceeding; those who did not consent were unable to continue. No personally identifiable information was collected, and all responses were anonymous.

### Statistical analysis

All statistical analyses were performed using Jamovi software (Version 2.7.18, Windows).

Descriptive statistics are reported as frequencies and percentages [n (%)] for categorical variables and as mean ± SD for continuous variables. The normality of continuous variables was assessed using the Shapiro–Wilk test. Both electronic cigarette knowledge scores (W=0.927, p<0.001) and HTPs knowledge scores (W=0.813, p<0.001) significantly deviated from a normal distribution. Therefore, non-parametric tests were used in subsequent analyses. Group comparisons according to intention to try HTPs (yes, no) were conducted using the Mann–Whitney U test, as HTP knowledge scores did not meet the assumptions of parametric testing. Effect sizes were reported using the rank-biserial correlation coefficient (r_rb). Associations between knowledge scores and Likert-type intention items were analyzed using Kendall’s tau-b (τ-b) correlation coefficient, given the ordinal nature of the variables.

Multivariable ordinal logistic regression analyses were performed to examine factors associated with intention to use electronic cigarettes and HTPs, treated as ordinal outcomes. Separate models were fitted for each intention outcome. Adjusted odds ratios (AORs) with 95% confidence intervals (CIs) were reported. Models were adjusted for age, sex, university type, conventional cigarette use, and peer use of the respective product. All tests were two-sided, and a significance level of α=0.05 was adopted.

## RESULTS

A total of 382 university students were included in the study. The mean age was 20.6 ± 3.9 years. The majority of participants were female (78.0%), and 52.5% were first-year students. Regarding conventional cigarette use, 62.2% reported never smoking, 18.1% reported occasional smoking, 15.7% reported daily smoking, and 3.9% reported having quit.

Awareness of electronic cigarettes was 94.2%. Ever use was reported by 35.9% of participants, past 30-day use by 5.0%, and current use by 0.8%. Awareness of HTPs was 34.8%. Ever use was reported by 13.6%, past 30-day use by 5.5%, and current use by 3.7%. Close friends’ use of electronic cigarettes was reported by 51.0% of participants, compared with 33.8% for HTPs (Table 1).

**Table 1:** Sociodemographic characteristics, product awareness and use among university students in Türkiye, 2025 (N=382)

*Variable*	*Category*	*n*	*%*
**Age** (years), mean ± SD		381	20.60 ± 3.92
**Sex**	Female	298	78.0
	Male	84	22.0
**Year of study**	1st	200	52.5
	2nd	156	40.9
	3rd	4	1.0
	4th	15	3.9
	5th	2	0.5
	Preparatory year	4	1.0
**Program type**	Associate degree	287	75.2
	Undergraduate degree	95	24.9
**University type**	Public	130	34.0
	Foundation (Private)	252	66.0
**Region of residence** (past 12 months)	Marmara	237	62.0
	Black Sea	71	18.6
	Central Anatolia	31	8.1
	Eastern Anatolia	17	4.5
	Southeastern Anatolia	12	3.1
	Mediterranean	8	2.1
	Aegean	6	1.6
**Conventional cigarette use**	Never	237	62.2
	Occasional	69	18.1
	Daily	60	15.7
	Former smoker	15	3.9
**Peer use of e-cigarettes**	Yes	195	51.0
	No	187	49.0
**Peer use of heated tobacco products** (HTPs)	Yes	129	33.8
	No	253	66.2
**Awareness of electronic cigarettes**	Yes	360	94.2
	No	22	5.8
**Awareness of heated tobacco products**	Yes	133	34.8
	No	249	65.2
**Ever used e-cigarettes**	Yes	137	35.9
	No	245	64.1
**Ever used HTPs**	Yes	52	13.6
	No	330	86.4
**Current e-cigarette use**	Yes, currently	3	0.8
	Tried but quit	124	32.5
	Never used	254	66.7
**Current HTP use**	Yes, currently	14	3.7
	Tried but quit	44	11.5
	Never used	323	84.8
**E-cigarette use** (past 30 days)	Yes	19	5.0
	No	362	95.0
**HTP use** (past 30 days)	Yes	21	5.5
	No	361	94.5
**I might consider trying e-cigarettes if offered by a friend**	Yes	42	11.6
	No	321	88.4
**I might consider trying HTPs if offered by a friend**	Yes	23	6.4
	No	338	93.6
**I might consider trying e-cigarettes in the future**	Yes	26	7.2
	No	336	92.8
**I might consider trying HTPs in the future.**	Yes	12	3.3
	No	349	96.7

Total n may vary across variables due to missing responses (e.g. age n=381).

The mean knowledge score was 3.25 ± 2.22 for electronic cigarettes (7 items) and 2.22 ± 2.51 for HTPs (8 Items). Knowledge scores for HTPs were compared according to intention to try HTPs using the Mann–Whitney U test. A statistically significant difference in HTP knowledge scores was observed between groups based on responses to the item ‘I might consider trying HTPs if offered by a friend’ (U=2978, p=0.047, r_rb= -0.234). The mean knowledge score was 3.00 ± 2.61 (median=3.00) among participants who responded ‘Yes’ and 1.94 ± 2.35 (median=1.00) among those who responded ‘No’. No statistically significant difference was found between groups based on responses to the item ‘I might consider trying HTPs in the future’ (p=0.129) (Table 2).

**Table 2:** Comparison of heated tobacco product (HTP) knowledge scores according to intention to try HTPs among university students in Türkiye, 2025 (N=382)

*Intention item*	*Group*	*n*	*Mean ± SD*	*Median*	*U*	*p*	*r_rb*
I might consider trying HTPs if offered by a friend	Yes	23	3.00 ± 2.61	3.00	2978	0.047*	-0.234
	No	338	1.94 ± 2.35	1.00			
I might consider trying HTPs in the future	Yes	12	2.92 ± 2.31	3.00	1584	0.129	-0.244
	No	349	1.98 ± 2.37	1.00			

Mann–Whitney U test was used. r_rb: rank-biserial correlation coefficient (effect size).

* p<0.05.

Associations between electronic cigarette knowledge scores and intention-to-use items were evaluated using Kendall’s tau-b correlation analysis. A weak but statistically significant positive correlation was observed between electronic cigarette knowledge scores and the item ‘I intend to use e-cigarettes within the next 6 months’ (τ-b=0.143, p<0.001). A similar weak positive correlation was found between electronic cigarette knowledge scores and the item ‘I may be inclined to use e-cigarettes’ (τ-b=0.165, p<0.001). In contrast, a strong positive correlation was observed between the two intention-to-use items (τ-b=0.784, p <0.001).

In multivariable ordinal logistic regression analyses, knowledge of electronic cigarettes was not significantly associated with intention to use e-cigarettes within the next 6 months (AOR=1.02; 95% CI: 0.91–1.14, p=0.721) or with the inclination to use e-cigarettes (AOR=1.08; 95% CI: 0.97–1.20, p=0.179). Conventional cigarette use was strongly associated with higher intentions in both models (AOR=6.50; 95% CI: 3.95–10.71; AOR= 6.38; 95% CI: 3.95–10.30; both p<0.001). Peer use of e-cigarettes was also significantly associated with increased intention, while age was inversely associated with intention in Model 1 (Table 3).

**Table 3:** Multivariable ordinal logistic regression analysis of factors associated with intention to use electronic cigarettes among university students in Türkiye, 2025 (N=382)

*Variable*	*Model 1: I intend to use e-cigarettes* *within the next 6 months* *AOR (95% CI)*	*p*	*Model 2: I may be inclined to use* *e-cigarettes* *AOR (95% CI)*	*p*
**Knowledge of e-cigarettes**	1.02 (0.91–1.14)	0.721	1.08 (0.97–1.20)	0.179
**Age** (years)	0.91 (0.84–0.98)	0.010**	0.94 (0.88–1.00)	0.055
**Sex** (Male)	1.13 (0.65–1.98)	0.670	0.84 (0.49–1.46)	0.539
**Conventional cigarette use**	6.50 (3.95–10.71)	<0.001***	6.38 (3.95–10.30)	<0.001***
**Peer use of e-cigarettes**	2.15 (1.33–3.47)	0.002**	1.74 (1.10–2.76)	0.018*
**University type** (private)	1.35 (0.81–2.23)	0.249	1.52 (0.93–2.48)	0.098

AOR: adjusted odds ratio. Models were adjusted for age, sex, university type, conventional cigarette use, and peer use of the respective product. Reference categories: female, non-smoker, public university.

* p<0.05,

** p<0.01,

*** p<0.001.

Similarly, knowledge of HTPs was not significantly associated with intention to use HTPs within the next 6 months. No statistically significant association was observed for inclination to use HTPs (p=0.053). Conventional cigarette use remained a strong predictor in both models (AOR=6.35 and AOR=6.11; both p<0.001). Male sex was associated with lower inclination to use HTPs (AOR=0.54, 95% CI: 0.30–0.97, p=0.040) (Table 4).

**Table 4:** Multivariable ordinal logistic regression analysis of factors associated with intention to use heated tobacco products among university students in Türkiye, 2025 (N=382)

*Variable*	*Model 3: I intend to use HTPs* *within the next 6 months* *AOR (95% CI)*	*p*	*Model 4: I may be inclined to* *use HTPs* *AOR (95% CI)*	*p*
**Knowledge of HTPs**	1.09 (0.99–1.19)	0.073	1.09 (1.00–1.20)	0.053
**Age** (years)	0.95 (0.89–1.01)	0.128	1.00 (0.94–1.06)	0.967
**Sex** (Male)	0.75 (0.42–1.34)	0.335	0.54 (0.30–0.97)	0.040*
**Conventional cigarette use**	6.35 (3.92–10.29)	<0.001***	6.11 (3.76–9.91)	<0.001***
**Peer use of HTPs**	1.45 (0.89–2.35)	0.134	1.01 (0.61–1.65)	0.983
**University type** (private)	1.51 (0.92–2.49)	0.104	1.26 (0.77–2.07)	0.352

AOR: adjusted odds ratio. Models were adjusted for age, sex, university type, conventional cigarette use, and peer use of the respective product. Reference categories: female, non-smoker, public university.

* p<0.05,

*** p<0.001.

## DISCUSSION

This study examined the association between university students’ knowledge of electronic cigarettes and HTPs and their intention to use these products. Awareness of electronic cigarettes was widespread, whereas familiarity with HTPs was comparatively lower. Participants demonstrated relatively low knowledge levels for both product types. In initial analyses, weak but statistically significant positive associations were observed between electronic cigarette knowledge and intention-to-use measures. Similarly, students who reported that they might consider trying HTPs if offered by a friend had higher HTP knowledge scores. However, in multivariable analyses, knowledge was not independently associated with intention to use either electronic cigarettes or HTPs. Conventional cigarette use emerged as the strongest and most consistent factor associated with intention to use both products, while peer e-cigarette use was also significantly associated with intention to use electronic cigarettes. These findings suggest that knowledge alone may not be sufficient to explain differences in young adults’ intentions to experiment with alternative tobacco products.

The World Health Organization (WHO) identifies young adults as a particularly vulnerable group with respect to emerging nicotine products and emphasizes that attitudes and perceptions formed during this developmental period may influence future patterns of use^15^. Given that the present study focused on university students within this age range, the findings highlight the importance of early preventive interventions targeting young adults.

The high awareness of electronic cigarettes observed in this study (94.2%) suggests that these products are widely recognized among university students. However, although 35.9% of participants reported ever trying e-cigarettes, current use was low (0.8%). This may indicate that a substantial proportion of those who tried the product did not continue regular use. Similarly, studies conducted among university students have reported relatively high rates of ever experimentation with electronic cigarettes, whereas daily use remains more limited^16,17^.

Findings related to HTPs present a different pattern from those observed for electronic cigarettes. The relatively low awareness rate (34.8%) may be associated with the later introduction of these products to the market and their comparatively more limited promotion compared with electronic cigarettes. In contrast, current use was higher for HTPs than for electronic cigarettes. This difference may reflect the perception that HTPs more closely resemble conventional cigarettes in terms of design and usage experience. Indeed, previous studies have reported that HTPs are perceived as more similar to conventional cigarettes than electronic cigarettes in terms of product characteristics and user experience^18^.

Although individual use of both product types was relatively low, more than half of participants reported peer use of electronic cigarettes (51.0%), and approximately one-third reported peer use of HTPs (33.8%). These findings suggest that university students may be frequently exposed to these products within their peer environment. Previous research indicates that nicotine product use among peers may reduce perceived risk and contribute to the normalization of such products^19,20^. Even if individual use has not yet become widespread, the social visibility of electronic cigarettes and HTPs within university peer networks may be associated with attitudes and intentions toward these products.

Knowledge levels of both electronic cigarettes and HTPs were relatively limited. The mean knowledge score was 3.25 ± 2.22 (maximum 7) for electronic cigarettes and 2.22 ± 2.51 (maximum 8) for HTPs. These scores suggest that knowledge about these products may be incomplete among young adults. Despite the high level of awareness of electronic cigarettes, this awareness does not necessarily correspond to comprehensive or accurate knowledge. This finding is consistent with previous studies reporting that, although awareness of electronic cigarettes is high, detailed knowledge regarding their contents, health effects, and addictive potential may remain limited among young adults^21,22^. Knowledge levels were higher for electronic cigarettes than for HTPs. This difference may reflect the longer market presence of electronic cigarettes, their greater media visibility, and their more frequent discussion among young adults^23^. In contrast, lower knowledge scores for HTPs may be related to their more recent introduction and comparatively lower public awareness. The lower level of knowledge regarding HTPs may suggest that perceptions of these products among young adults are not fully aligned with scientific evidence^3^. Marketing messages that frame HTPs as ‘less harmful’ or as ‘alternatives’ to conventional cigarettes may contribute to favorable perceptions, independent of detailed knowledge. In a study conducted among young adults in the United States, awareness and use of HTPs were relatively low, yet perceptions of harm and addiction potential were comparatively favorable^10^. This pattern suggests that limited knowledge and favorable perceptions may coexist, and that young adults may form evaluations of HTPs without comprehensive scientific understanding.

A statistically significant association was observed between knowledge level and intention to try HTPs. Participants who indicated that they might consider trying HTPs if offered by a friend had higher knowledge scores. Previous studies have reported that, among young adults, perceptions and knowledge regarding HTPs are often framed around narratives portraying these products as ‘less harmful’ than combustible cigarettes; such favorable and selective perceptions have been found to be associated with experimentation tendencies and perceived social acceptability^3,10^. Furthermore, reports from the WHO emphasize that limited and selective communication about newly introduced and technologically framed nicotine products may reduce risk perception and may be associated with increased curiosity and experimentation^4,24^. In contrast, no statistically significant difference in knowledge scores was observed for the item ‘I might consider trying HTPs in the future’. This finding suggests that knowledge level may not be equally associated with all indicators of intention and that intention may vary depending on contextual framing. Within the framework of the theory of planned behavior, behavioral intention is understood to be influenced not only by cognitive factors but also by situational elements such as perceived social norms and perceived behavioral control^13^. In this context, an intention item that includes a concrete social cue – such as an offer from a friend – may show a stronger association with knowledge level.

For electronic cigarettes, low but statistically significant positive correlations were found between the knowledge score and the intention-to-use items. Given the weak correlations, knowledge appears to play a limited role in relation to intention to use. The literature presents differing findings on this relationship. These inconsistencies across studies may reflect differences in study populations, measurement tools used to assess knowledge and intention, sample size, and sociocultural context. In one study, knowledge about electronic cigarettes was reported to have a direct, positive, and strong association with intention to use. Increasing knowledge did not discourage individuals from using e-cigarettes; rather, together with social influence and health perceptions, it was associated with a stronger intention to use^25^. In another study, however, knowledge level was not directly associated with intention. Instead, attitudes and social norms were found to have a significant influence on intention^26^.

Multivariable analyses revealed that knowledge of electronic cigarettes was not independently associated with intention to use e-cigarettes, despite weak associations observed in initial analyses. This finding suggests that the relationship between knowledge and intention may be influenced by other behavioral and social factors. In contrast, conventional cigarette use emerged as the strongest factor associated with intention to use e-cigarettes. Additionally, peer use of e-cigarettes was found to be significantly associated with intention, underscoring the role of social influence in shaping tobacco-related behaviors among young adults. Evidence from a systematic review indicates that peer use and social norms are consistent predictors of intention to use e-cigarettes^27^, while longitudinal findings further demonstrate that young adults with peers who use e-cigarettes are significantly more likely to initiate use themselves^28^.

Similarly, knowledge was not independently associated with intention to use HTPs within the next 6 months or inclination to use them, while conventional cigarette use was the strongest and most consistent factor associated with both outcomes. This suggests that the intention to use HTPs appears to be more strongly associated with existing smoking behavior than with knowledge alone. Previous research has demonstrated that HTP use is significantly more prevalent among current and former smokers^29,30^, indicating that these products are more commonly used by individuals who already use tobacco. Male sex was associated with lower inclination to use HTPs in one model, which may indicate potential gender differences in perceptions or social acceptability. Unlike e-cigarettes, peer use of HTPs was not significantly associated with intention, which may reflect the lower awareness and social visibility of these products among young adults.

### Strengths and limitations

The present study contributes to the limited evidence on knowledge and intention related to electronic cigarettes and HTPs among university students in Türkiye. By examining both product types within the same sample, the study provides a comparative perspective on awareness, knowledge, and behavioral intention. The inclusion of both knowledge measures and intention-to-use indicators allowed for a more comprehensive evaluation of cognitive and behavioral dimensions. The use of non-parametric statistical tests and the reporting of effect sizes strengthen the transparency of the analytical approach. In addition, the use of multivariable regression analyses allowed for the consideration of potential confounding factors, which may help in the interpretation of the findings.

However, certain limitations should be acknowledged. The cross-sectional design precludes conclusions about causality between knowledge and intention to use. The use of non-probability convenience sampling may have introduced selection bias and may limit the generalizability of the findings to the broader population of university students.

Data were self-reported and may therefore be subject to recall bias and social desirability bias. Additionally, participants who completed less than 80% of the questionnaire were excluded from the analysis, which may have introduced selection bias. The sample was also predominantly female, which may limit the generalizability of the findings. Furthermore, knowledge was assessed using a limited number of items; future research may benefit from more comprehensive instruments that also capture harm perception, risk beliefs, and social normative influences. Future studies with larger prospective designs may help to clarify the temporal relationship between knowledge and intention.

## CONCLUSIONS

Knowledge about electronic cigarettes and HTPs was not independently associated with intention to use in multivariable analyses. Knowledge alone may therefore not be sufficient to explain differences in intentions among young adults. Behavioral and social factors, such as conventional cigarette use and peer influence, appear to be more strongly associated with intentions to use these products. Exposure to product-related information that does not clearly communicate risks may be associated with more favorable perceptions. These findings suggest that preventive strategies may consider factors beyond information provision alone, including risk perception, misconceptions, and social influences.

## Supplementary Material



## Data Availability

The data supporting this research are available from the author on reasonable request.
